# Supplementary figures and images for: Modelling indoor airborne transmission combining architectural design and people movement using the VIRIS simulator and web app
Source: Sci Rep. 2024 Nov 15;14:28220. doi: 10.1038/s41598-024-79525-6 (PMC11568127; doi:10.1038/s41598-024-79525-6)

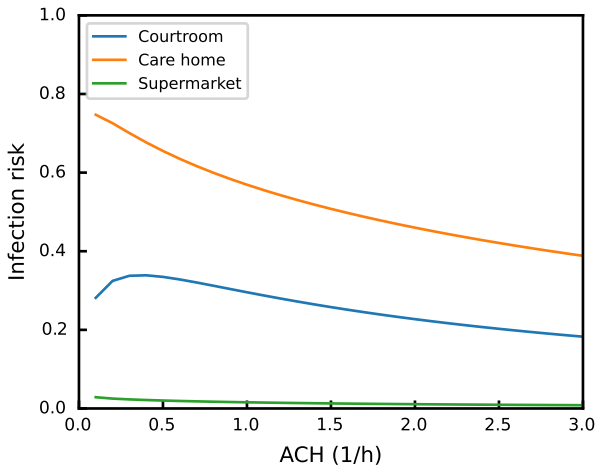

Supplement: Supplementary file 1 — Supplementary Information. [file 41598_2024_79525_MOESM1_ESM.pdf]
